# Lower respiratory tract infection and rapid expansion of an abdominal aortic aneurysm: a case report

**DOI:** 10.1186/1752-1947-4-333

**Published:** 2010-10-21

**Authors:** Steven Naylor, Zakareya Gamie, Ravinder S Vohra, Sapna Puppala, Patrick J Kent, D Julian A Scott

**Affiliations:** 1 The Leeds Vascular Institute, The General Infirmary at Leeds, Great George Street, Leeds LS1 3EX, UK; 2 Department of Interventional Radiology, The General Infirmary at Leeds, Great George Street, Leeds LS1 3EX, UK; 3 Division of Cardiovascular and Diabetes Research, Leeds Institute of Genetics, Health and Therapeutics, University of Leeds, Clarendon Way, Leeds LS2 9JT, UK

## Abstract

**Introduction:**

The rate of abdominal aortic aneurysm expansion is related to multiple factors. There is some evidence that inflammation can accelerate aneurysm expansion. However, the association between pulmonary sepsis and rapid abdominal aortic aneurysm expansion is rarely reported.

**Case presentation:**

Here we present a case of a rapidly expanding abdominal aortic aneurysm in a 68-year-old Caucasian man with a concomitant lower respiratory tract infection and systemic sepsis requiring intensive monitoring and urgent endovascular intervention. Our patient had an uncomplicated post-operative recovery and a follow-up computed tomography scan at one month demonstrated no evidence of an endoleak.

**Conclusion:**

This case highlights the potential association between pulmonary sepsis and rapid abdominal aortic aneurysm expansion. In such cases, a policy of frequent monitoring should be adopted to identify those patients requiring definitive management.

## Introduction

Studies suggest that an abdominal aortic aneurysm (AAA) expands on average at 0.25 cm per annum [1]. This is proportional to the size of the AAA [2,3], and has been linked to factors such as smoking, hypertension, advanced age and cardiac disease [4]. There are rare reports that aneurysmal disease can expand in the presence of lung sepsis over a few months [5,6] . The presence of pulmonary disease may increase inflammatory mediators and result in weakening of the aortic wall [5]. Here we report the case of a sudden expansion of an infra-renal AAA in a patient with a lower respiratory tract infection (LRTI) and sepsis.

## Case presentation

**Figure 1:**
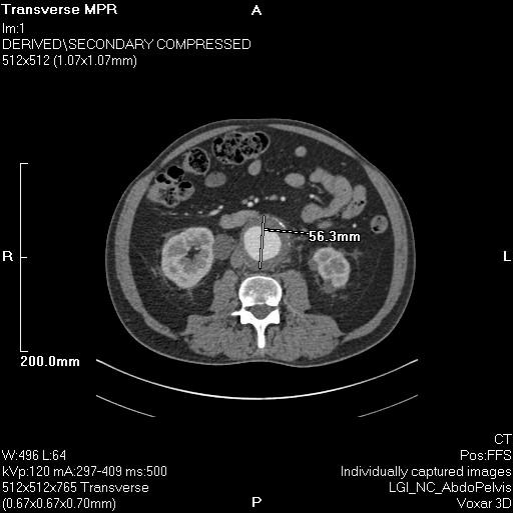
**CT aortogram demonstrating a 5.6 cm anteroposterior diameter AAA**.

**Figure 2:**
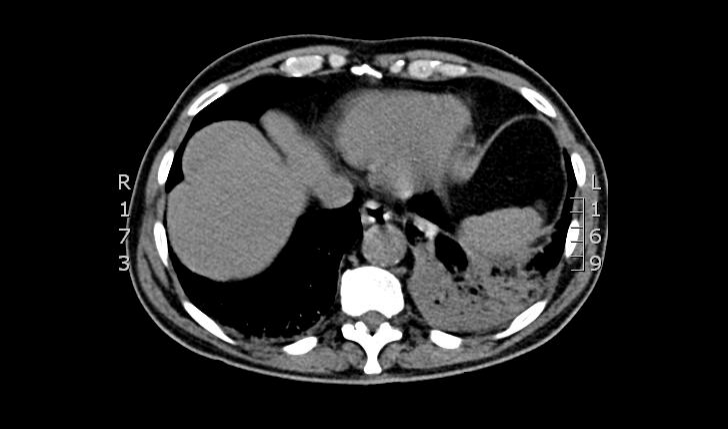
**CT thorax demonstrating extensive lower lobe consolidation and collapse noted in the left lung with extensive hilar lymphadenopathy**.

A 68-year-old Caucasian man with a known infra-renal AAA was admitted with shortness of breath, a presumed community acquired LRTI and increasing back pain and epigastric discomfort. His past medical history included ischemic heart disease and he underwent a coronary artery bypass graft in 1987. He smoked approximately 20 cigarettes per day for 20 years. The AAA had been under six-monthly surveillance since 2005. At that time, the maximum diameter was 4.9 cm and it had grown to 5.2 cm over a period of one year. The most recent ultrasound scan was performed two months prior to admission and at that time the maximal diameter of the AAA was 5.4 cm. Our patient was hemodynamically stable, but pyrexial and hypoxic. Clinical examination revealed signs of a left bronchopneumonia and tender epigastrium. A leukocytosis with neutrophilia of 15.03 × 10^9^/L was demonstrated on his blood investigations. An urgent computed tomography (CT) aortogram confirmed a non-leaking 5.6 cm AAA (Figure 1). In addition, extensive lower lobe consolidation and collapse with hilar lymphadenopathy was noted (Figure 2). A diagnosis of a lobar pneumonia was made. However, the tenderness in the epigastrium was a concern and could represent either a symptomatic aneurysm or referred pain from the lobar pneumonia. Thus, our patient was closely observed and the pneumonia treated aggressively with intravenous antibiotics, supplemental oxygen and physiotherapy.

**Figure 3:**
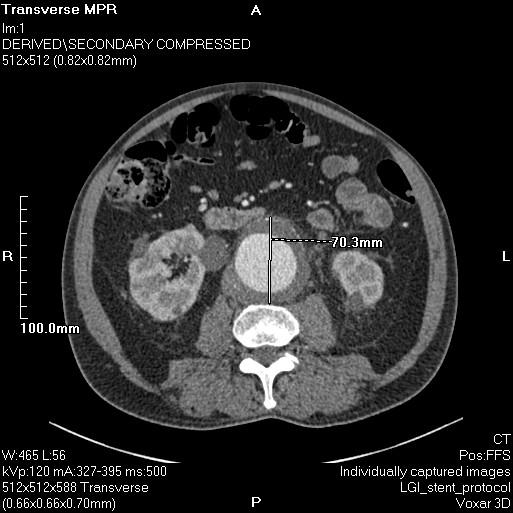
**CT aortogram demonstrating a rapid increase in the size of the AAA measuring 7.0 cm in the anteroposterior diameter**. There is a new beak in the left lateral aortic thrombus and signs of impending rupture.

**Figure 4:**
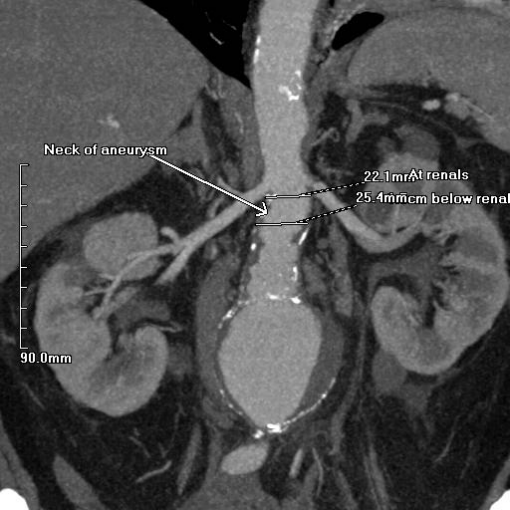
**Coronal CT angiogram image demonstrating the 7.0 cm aneurysm**. The neck of the aneurysm was not angulated and its diameter at the renal arteries was 22.1 mm and below the renal arteries was 25.4 mm.

Forty-eight hours following admission our patient reported symptoms of pre-syncope with a brief period of hypotension. A repeat CT aortogram demonstrated a rapid increase in size of the AAA to 7.0 cm and retroperitoneal fat stranding (Figures 3 and 4). The neck of the aneurysm did not show any significant angulation and its juxta-renal diameter was 22.1 mm increasing to 25.4 mm in its infra-renal segment. In addition, there was a significant stenosis of the left common iliac artery.

Despite aggressive treatment of our patient's pneumonia, he remained hypoxic. The options were either an emergency open bifurcated aortic graft or an endovascular aorto-uni-iliac repair with a femoral-to-femoral cross-over procedure. Following a full discussion with our patient, anesthetists and endovascular radiologists, the latter procedure was performed. Our patient had an uncomplicated post-operative recovery. He was continued on intravenous antibiotics for a further five days and discharged. He was followed up clinically at four, five and seven months post-operatively. CT scans at one and six months post-operatively showed good stent position and patency.

## Discussion

The expansion rate of AAAs varies according to numerous factors. The probability of rupture of a 5 cm and 7 cm AAA is less than 16% and 25% per year, respectively [7] and guidelines in the United Kingdom recommend three-monthly assessment with an abdominal ultrasound for AAAs greater than 5 cm [8]. In small AAAs with a size of 3.0 to 3.9 cm growth, the growth rate has been reported as an average 0.11 cm annually [2]. AAAs with a diameter of between 4.0 and 4.9 cm have been found to have a much larger rate of growth with an average rate of 0.79 cm per year in those with continuous expansion compared to 0.27 cm per year with discontinuous (staccato) expansion [3]. Thus the typical expansion rate is about 0.25 cm per annum, and if the aneurysm diameter increases by 0.4 to 0.8 cm per year more frequent surveillance is recommended [9]. AAA expansion varies individually and inflammation can influence this process and dramatically accelerate AAA expansion as a result of specific cellular immune responses [10,11].

In the case presented here, the AAA had increased in size by about 0.3 cm per annum until admission. In the presence of concomitant sepsis it suddenly expanded. In the wall of an AAA there is up-regulation of pro-inflammatory IL-1β, IL-6, IL-10 and TNF-α, which have been shown to positively correlate with aneurysm growth [12,13]. Such cytokines, chemokines and growth factors are known to be further potentiated during septic events such as a LRTI [14]. One possibility is that concomitant sepsis could increase these specific inflammatory mediators within the AAA wall further weakening the aortic wall, increasing the risk of expansion and rupture.

There is a documented association between ongoing pulmonary sepsis, expansion of aortic aneurysms and aortic dissection [5,15]. This has been more commonly reported in thoracic than AAAs. However, hematogenous seeding may also affect the abdominal aorta, if there is no contiguous focus of infection [6]. The expansion and change in fat around the AAA found on the repeat CT aortogram suggested inflammation or an impending leak. In the case presented here, it is not clear whether this represented a mycotic AAA. However, the fusiform nature of the pre-existing AAA and lack of air in the aneurysm sac do not support a mycotic AAA. Regardless, there is controversy regarding the use of endovascular approaches in such aneurysms; however, there are several reports which demonstrate better outcomes when compared to conventional surgery in these high risk cases [16]. Despite these concerns we proceeded with an endovascular repair, which was uneventful.

## Conclusions

This case highlights the potential association between pulmonary sepsis and rapid AAA expansion. In these patients there must be a high index of clinical suspicion for rapid progression, and a policy of frequent monitoring may be adopted to identify those patients requiring definitive management. Endovascular repair may be suitable in certain cases depending on aneurysm morphology and local experience.

## Abbreviations

AAA: abdominal aortic aneurysm; CT: computed tomography; LRTI: lower respiratory tract infection.

## Competing interests

The authors declare that they have no competing interests.

## Consent

Written informed consent was obtained from the patient for publication of this case report and any accompanying images. A copy of the written consent is available for review by the Editor-in-Chief of this journal.

## Authors' contributions

SN reviewed the literature and wrote a first draft of the manuscript. ZG reviewed the literature, corrected, finalized and submitted the manuscript. RSV reviewed the literature and was involved in manuscript preparation and editing. SP interpreted the radiological images and performed the endovascular stent procedure. PJK carried out the surgical procedure and was involved with manuscript editing and reviewing. DJAS was involved with the conception of the report and was involved with the surgical procedure. All authors read and approved the final manuscript.
